# Apparent Diffusion Coefficient for Distinguishing Between Malignant and Benign Lesions in the Head and Neck Region: A Systematic Review and Meta-Analysis

**DOI:** 10.3389/fonc.2019.01362

**Published:** 2020-01-08

**Authors:** Alexey Surov, Hans Jonas Meyer, Andreas Wienke

**Affiliations:** ^1^Department of Diagnostic and Interventional Radiology, University of Leipzig, Leipzig, Germany; ^2^Institute of Medical Epidemiology, Biostatistics and Informatics, Martin-Luther-University Halle-Wittenberg, Halle, Germany

**Keywords:** head and neck, tumors, apparent diffusion coefficient, ADC, magnetic resonance imaging, MRI

## Abstract

**Background:** The purpose of the present meta-analysis was to provide evident data about use of apparent diffusion coefficient (ADC) values for distinguishing malignant and benign lesions in the head and neck region.

**Material and Methods:** MEDLINE and Scopus databases were screened for associations between ADC and malignancy/benignancy of head and neck lesions up to December 2018. Overall, 22 studies met the inclusion criteria. The following data were extracted: authors, year of publication, study design, number of patients/lesions, lesion type, mean value, and standard deviation of ADC. The primary endpoint of the systematic review was the analysis of the association between lesion nature and ADC values. The methodological quality of the involved studies was checked according to the Quality Assessment of Diagnostic Accuracy Studies (QUADAS) instrument. The meta-analysis was undertaken by using RevMan 5.3 software. DerSimonian and Laird random-effects models with inverse-variance weights were used without further correction to account for the heterogeneity between the studies. Mean ADC values including 95% confidence intervals were calculated separately for benign and malignant lesions.

**Results:** The acquired 22 studies comprised 1,227 lesions. Different malignant lesions were diagnosed in 818 cases (66.7%) and benign lesions in 409 cases (33.3%). The mean ADC value of the malignant lesions was 1.04 × 10^−3^ mm^2^/s, and the mean value of the benign lesions was 1.46 × 10^−3^ mm^2^/s. Lymphomas and sarcomas showed the lowest calculated mean ADC values, 0.7 and 0.79 × 10^−3^ mm^2^/s, respectively. Adenoid cystic carcinomas had the highest ADC values (1.5 × 10^−3^ mm^2^/s). None of the analyzed malignant tumors had mean ADC values above 1.75 × 10^−3^ mm^2^/s.

**Conclusion:** ADC values play a limited role in distinguishing between malignant and benign lesions in the head and neck region. It may be only suggested that lesions with mean ADC values above 1.75 × 10^−3^ mm^2^/s are probably benign. Further large studies are needed for the analysis of the role of diffusion-weighted imaging (DWI)/ADC in the discrimination of benign and malignant lesions in the head and neck region.

## Introduction

Diffusion-weighted imaging (DWI) is a magnetic resonance imaging (MRI) technique based on measure of water diffusion in tissues (1). Restriction of water diffusion can be quantified by apparent diffusion coefficient (ADC) (1). Numerous studies have reported that DWI/ADC can provide information regarding histological architecture of tissues. According to the literature, ADC is associated with several histopathological features, such as cell count and expression of proliferation markers (2, 3). So it has been shown that ADC correlated well with expression of Ki67 in head and neck squamous cell carcinoma (4, 5). Furthermore, ADC can predict other important histopathological features, such as expression of vascular endothelial growth factor, tumor suppressor protein p53, hypoxia-inducible factor (HIF)-1α, CD3-positive cell count, and human papilloma virus (p16) (5–7).

In clinical setting, however, a key question is whether DWI/ADC can be used for distinguishing between malignant and benign lesions. Overall, it is well-known that malignant tumors have lower ADC values than have benign lesions. However, the physician needs plausible threshold values in his or her daily practice. Previously, some reports analyzed the diagnostic potential of DWI in the head and neck region (HNR). However, most reported studies investigated relatively small samples of up to 100 patients/lesions, and, therefore, the provided data cannot be applied as evident. Furthermore, the reported studies provided a broad spectrum of ADC threshold values. For example, Wang et al., based on an analysis of 97 different head and neck lesions, proposed a diagnostic scale of ADC values to predict malignancy in HNR (8). It has been shown that ADC values ≤0.65 × 10^−3^ mm^2^/s had a positive predictive value of malignancy of 100% and that ADC values ≤1.01 × 10^−3^ mm^2^/s had a positive predictive value of malignancy of 90% (8). In the study of Das et al. investigating 79 sinonasal masses, a cutoff ADC value of 1.791 × 10^−3^ mm^2^/s was identified to differentiate malignant and benign lesions with a sensitivity of 80% and specificity of 83.3% (9). Finally, Li et al. studied 78 patients with lingual lesions and calculated a threshold ADC value of <1.31 × 10^−3^ mm^2^/s (sensitivity, 92.6%; specificity, 97.3%) (10).

The aim of the present meta-analysis was to provide data regarding use of ADC for distinguishing malignant and benign lesions in the HNR based on a large sample.

## Materials and Methods

### Data Acquisition and Proving

**Figure 1:**
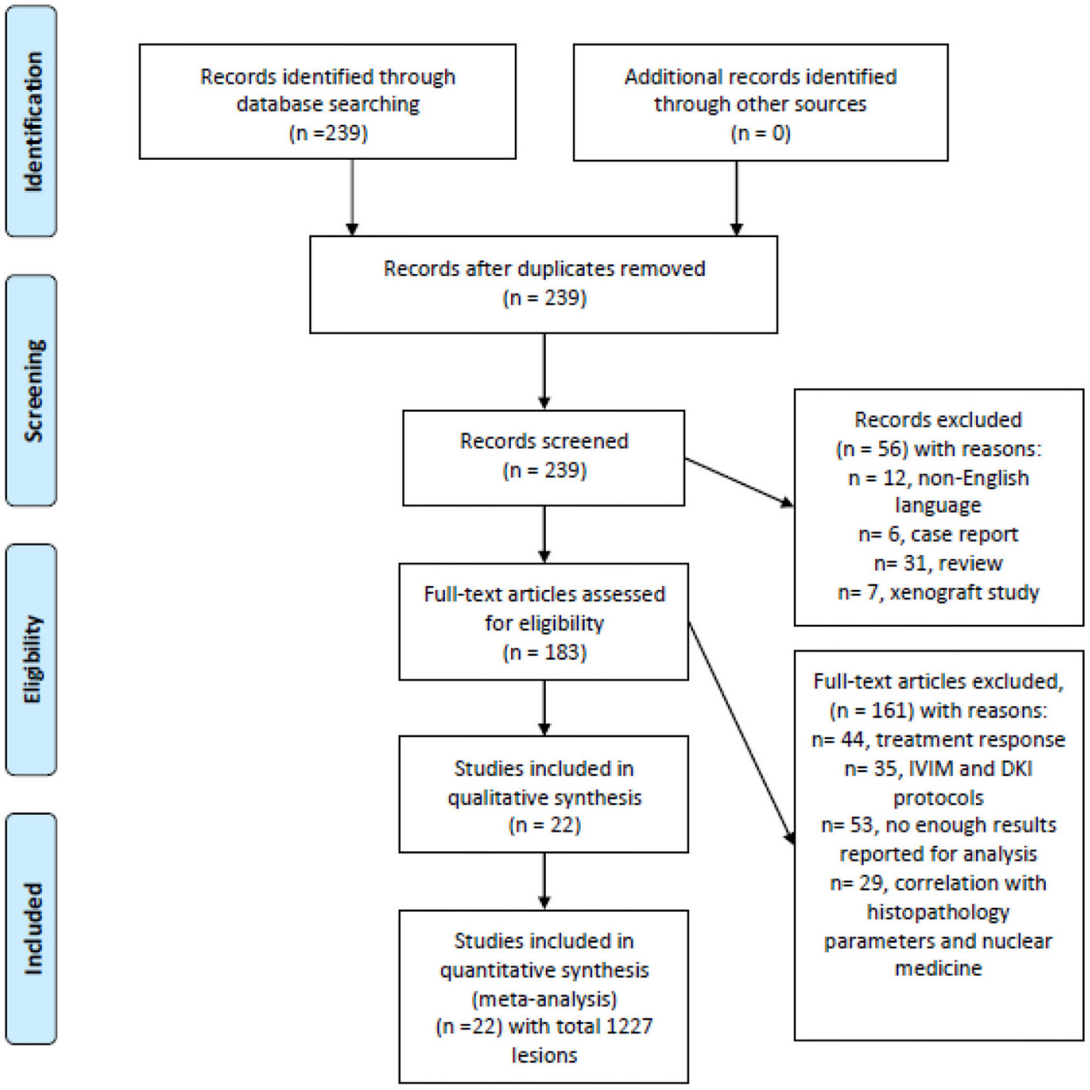
Preferred reporting items for systematic reviews and meta-analyses (PRISMA) flowchart of the data acquisition.

MEDLINE and Scopus databases were screened for associations between ADC and malignancy/benignancy of head and neck lesions up to December 2018 (Figure 1). The search terms/combinations were as follows:

“DWI or diffusion weighted imaging or diffusion-weighted imaging or ADC or apparent diffusion coefficient or DWI or diffusion weighted imaging AND head and neck OR neck carcinoma OR neck cancer OR neck neoplasm OR neck tumor.” Secondary references were also manually checked. The Preferred Reporting Items for Systematic Reviews and Meta-Analyses (PRISMA) statement was used for the research (11).

The primary endpoint of the systematic review was the analysis of association between the nature of head and neck lesions and their ADC values. The primary search identified 239 records. The abstracts of the items were checked. Inclusion criteria for this work were as follows:

- data regarding ADC derived from DWI,
- available mean and standard deviation values of ADC,
- original studies that investigated humans, and
- written in English.

Overall, 22 studies meet the inclusion criteria (9, 10, 12–31). Other 217 records were excluded from the analysis. Exclusion criteria were as follows:

- studies unrelated to the research subjects,
- studies with incomplete data,
- not written in English,
- duplicate publications,
- experimental animal and *in vitro* studies, and
- review, meta-analysis, and case report articles.

On the next step, the following data were extracted from the literature: authors, year of publication, study design, number of patients/tumors, tumor/lesion type, and mean value and standard deviation of ADC.

### Meta-Analysis

**Figure 2:**
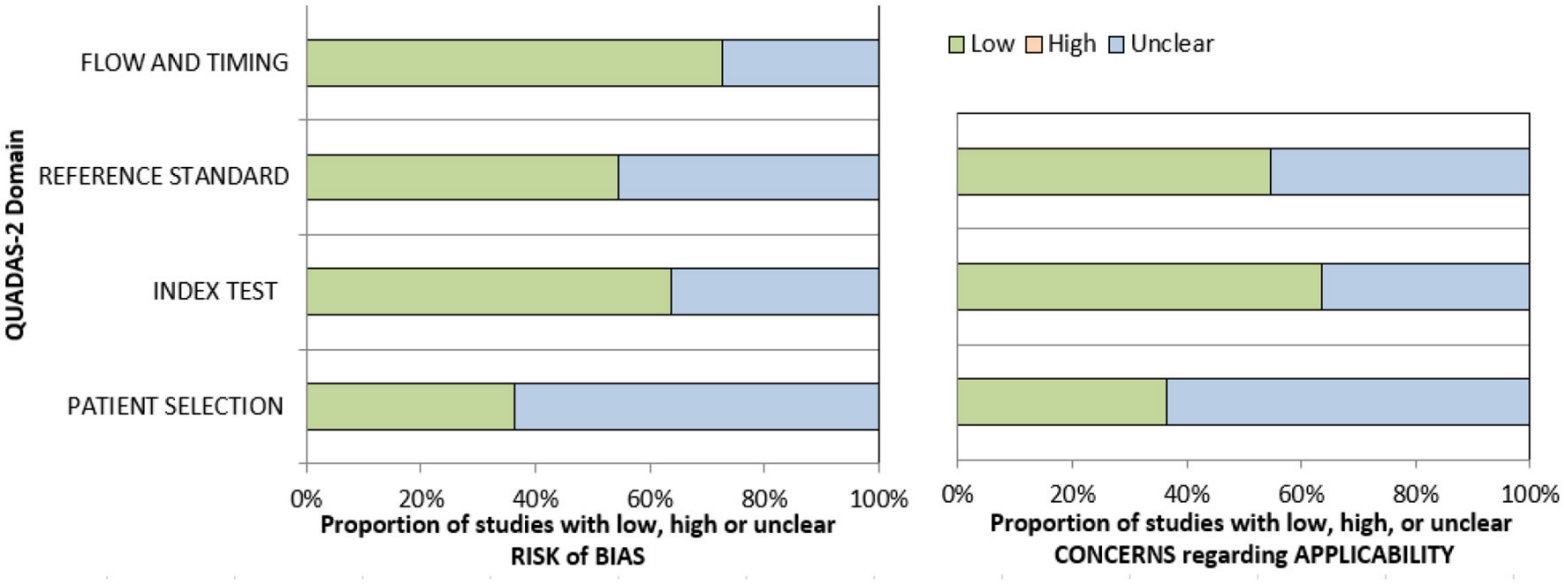
Quality assessment of diagnostic accuracy studies (QUADAS)-2 quality assessment of the included studies.

The methodological quality of the identified 22 studies was checked according to the Quality Assessment of Diagnostic Accuracy Studies (QUADAS) instrument (32) independently by two observers (A.S. and H.J.M.) (Figure 2).

The meta-analysis was undertaken by using RevMan (RevMan 2014, the Cochrane Collaboration Review Manager Version 5.3). Heterogeneity was calculated by means of the inconsistency index *I*^2^ (33, 34). DerSimonian and Laird random-effects models with inverse-variance weights were used without corrections (35). Mean ADC values including 95% confidence intervals were calculated separately for benign and malignant lesions.

## Results

**Figure 3:**
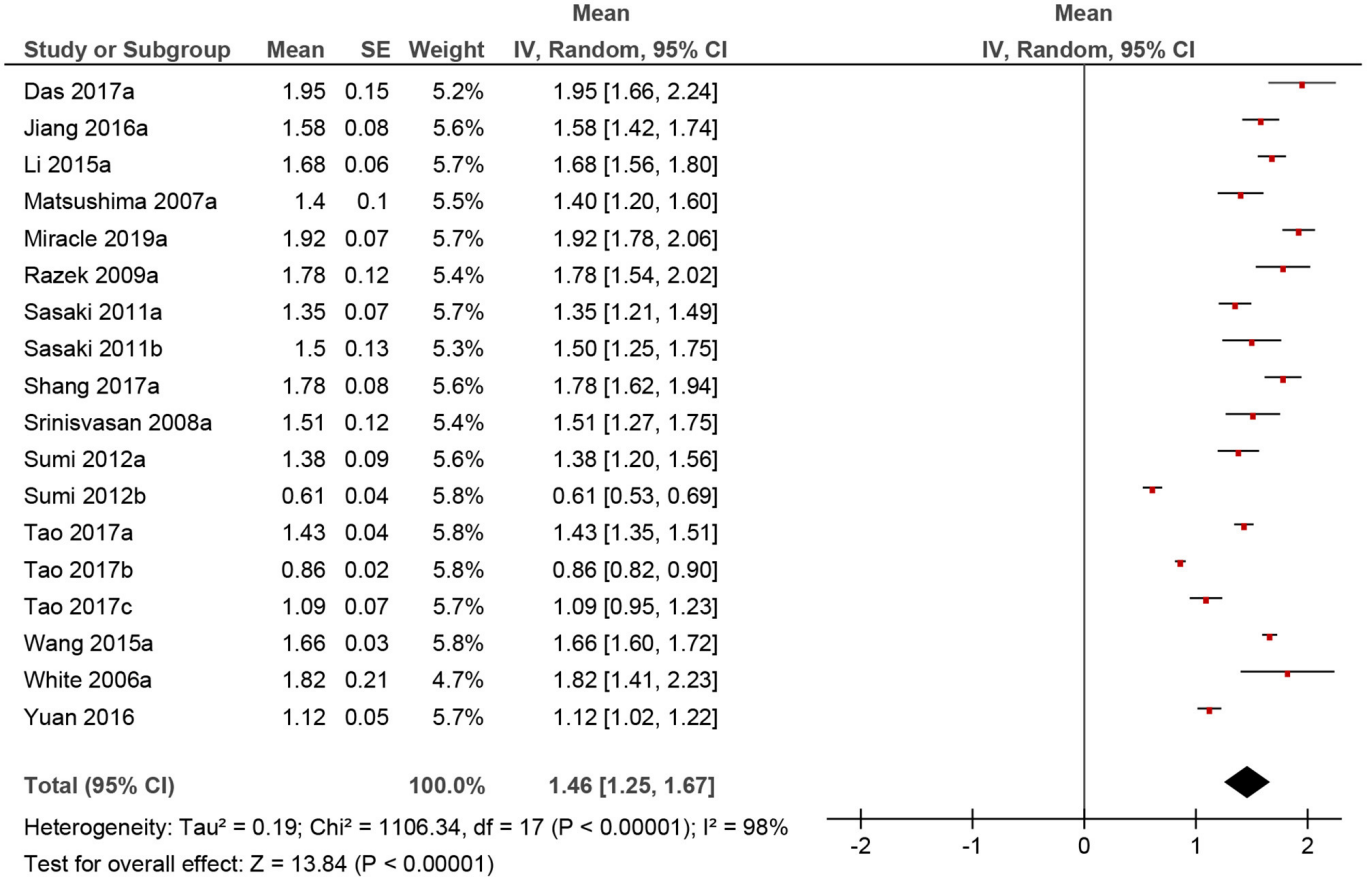
Forrest plots of apparent diffusion coefficient (ADC) values of benign lesions of the head and neck region.

Of the included 22 studies, 14 (64%) were retrospective, and 8 (36%) prospective. Overall, these studies comprised 1,227 lesions. Different malignancies of the HNR were diagnosed in 818 cases (66.7%) and benign lesions in 409 cases (33.3%) (Table 1). The mean ADC values of the malignant lesions ranged from 0.75 to 1.35 × 10^−3^ mm^2^/s, and the calculated mean value was 1.04 × 10^−3^ mm^2^/s (Figure 3).

**Table 1:** Malignant tumors and benign lesions involved in the analysis.

**Malignant tumors**	***n* (%)**
Squamous cell carcinoma	485 (59.3)
Lymphoma	87 (10.6)
Adenoid cystic carcinoma	40 (4.9)
Rhabdomyosarcoma	23 (2.8)
Malignant melanoma	23 (2.8)
Undifferentiated carcinoma	22 (2.7)
Olfactory neuroblastoma	22 (2.7)
Mucoepidermoid carcinoma	15 (1.8)
Adenocarcinoma	12 (1.5)
Unclassified sarcoma	10 (1.2)
Inverted papilloma with malignant transformation	10 (1.2)
Metastasis	7 (0.9)
Lymphoepithelial carcinoma	6 (0.7)
Malignant pleomorphic carcinoma	6 (0.7)
Plasmacytoma	5 (0.6)
Carcinoma ex pleomorphic adenoma	5 (0.6)
Osteosarcoma	4 (0.5)
Ewing's sarcoma	4 (0.5)
Acinic cell carcinoma	4 (0.5)
Chondrosarcoma	3 (0.4)
Sinonasal neuroendocrine carcinoma	3 (0.4)
Primitive neuroectodermal tumor	2 (0.2)
Salivary duct carcinoma	2 (0.2)
Malignant fibrous histiocytoma	2 (0.2)
Spindle cell sarcoma	2 (0.2)
Epi-myo-epi carcinoma	1 (0.1)
Myoepithelial carcinoma	1 (0.1)
Low-grade myxoid sarcoma	1 (0.1)
Myxoid liposarcoma	1 (0.1)
Malignant peripheral nerve sheath tumor	1 (0.1)
Adenosquamous cell carcinoma	1 (0.1)
Esthesioneuroblastoma	1 (0.1)
Transitional carcinoma	1 (0.1)
Papillary cystadenocarcinoma	1 (0.1)
Trichilemmal carcinoma	1 (0.1)
Leiomyosarcoma	1 (0.1)
Malignant hemangiopericytoma	1 (0.1)
Esthesioneuroblastoma	1 (0.1)
Transitional carcinoma	1 (0.1)
Total	818 (100)
**Nonmalignant lesions**	***n*** **(%)**
Pleomorphic adenoma	72 (17.6)
Inverted papilloma	63 (15.4)
Warthin's tumor	53 (13.0)
Inflammatory polyp	35 (8.6)
Juvenile nasopharyngeal angiofibroma	23 (5.6)
Vascular malformation	18 (4.4)
Precancerous laryngeal lesion	17 (4.2)
Cyst	12 (2.9)
Hemangioma	12 (2.9)
Acute rhinosinusitis	12 (2.9)
Schwannoma	12 (2.9)
Paraganglioma	10 (2.4)
Acute invasive fungal sinusitis	8 (2.0)
Basal cell adenoma	6 (1.5)
Ossifying fibroma	5 (1.2)
Organized hematoma	5 (1.2)
Meningioma	4 (1.0)
Fibroangioma	4 (1.0)
Chronic sinusitis	4 (1.0)
Chronic fungal sinusitis	4 (1.0)
Oncocytoma	3 (0.7)
Aneurysmal bone cyst	3 (0.7)
Spindle cell tumor	3 (0.7)
Lipomatous hemangiopericytoma	3 (0.7)
Neurofibroma	2 (0.5)
Benign ameloblastoma	2 (0.5)
Glomus jugulare	2 (0.5)
Myoepithelioma	2 (0.5)
Fibrous tumor of bone	2 (0.5)
Fibrous dysplasia	1 (0.2)
Sarcoidosis	1 (0.2)
Mucocele	1 (0.2)
Mesenchymal proliferation	1 (0.2)
Sphenoid pituitary adenoma	1 (0.2)
Hamartoma	1 (0.2)
Lingual thyroid	1 (0.2)
Enamel cell tumor	1 (0.2)
Total	409 (100)

**Figure 4:**
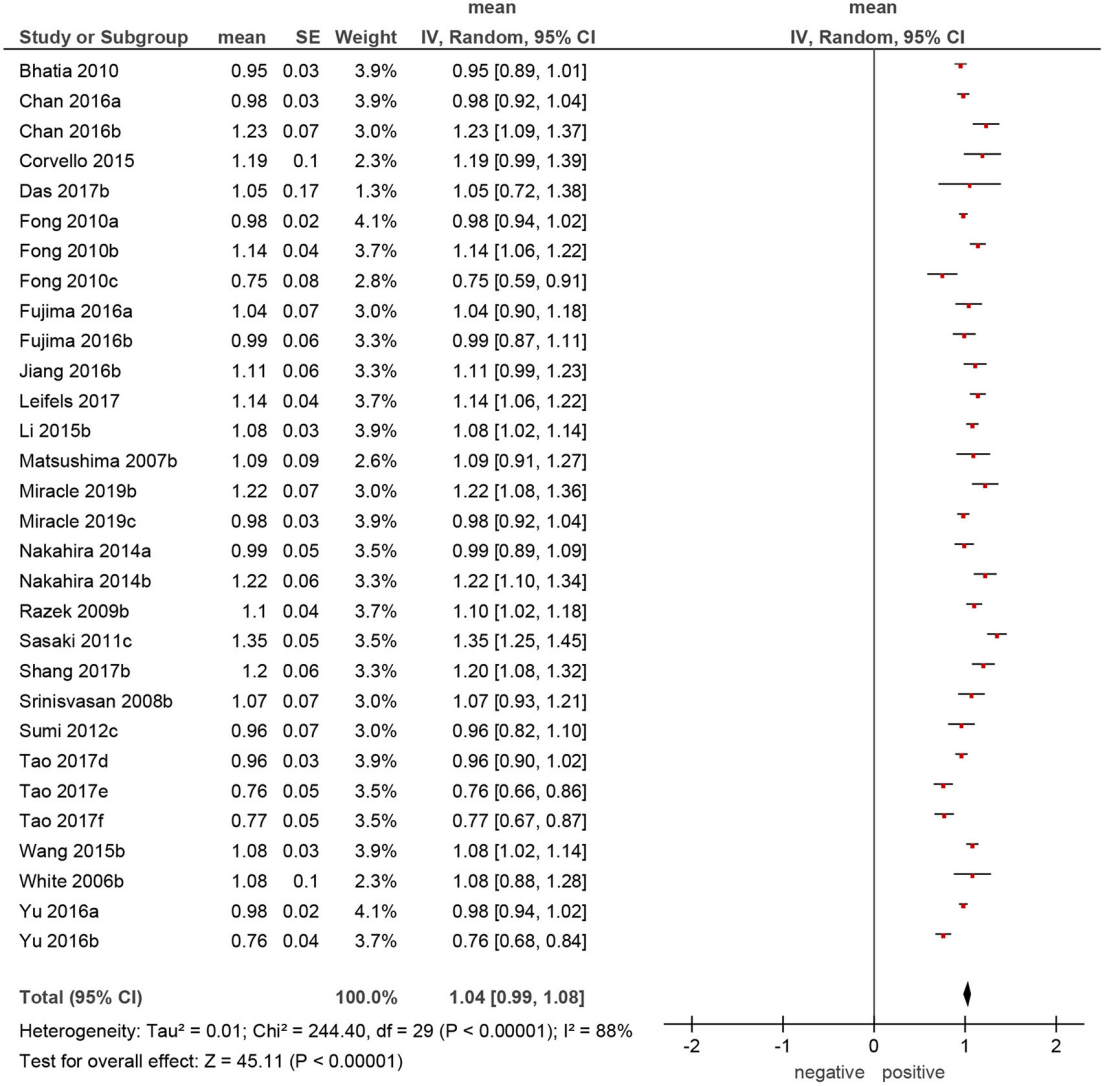
Forrest plots of apparent diffusion coefficient (ADC) values of malignant tumors of the head and neck region.

**Figure 5:**
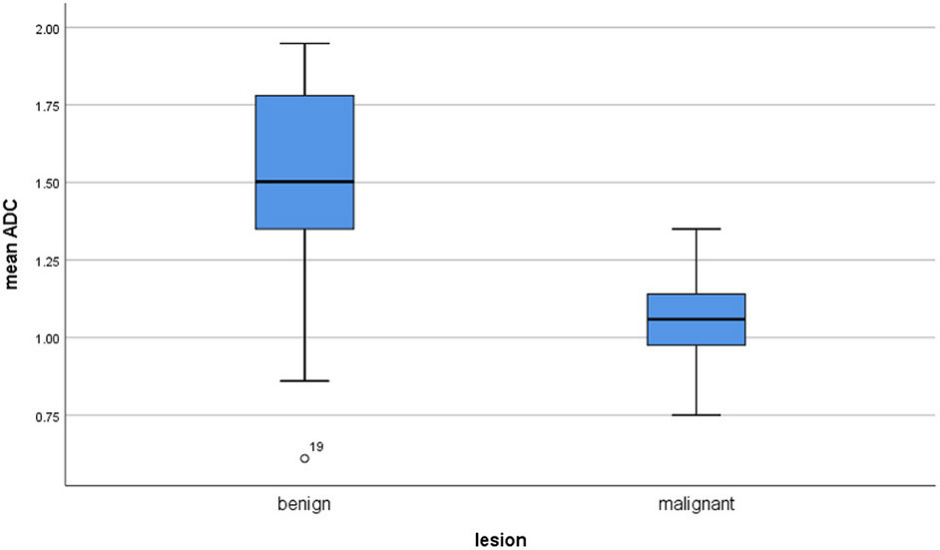
Comparison of apparent diffusion coefficient (ADC) values between malignant and benign lesions.

The calculated mean value of the benign lesions was 1.46 × 10^−3^ mm^2^/s, and the range of the collected ADC values was 0.61–1.95 × 10^−3^ mm^2^/s (Figure 4). The graphical distribution of ADC values in malignant and benign lesions is shown in Figure 5. The ADC values overlapped significantly.

**Figure 6:**
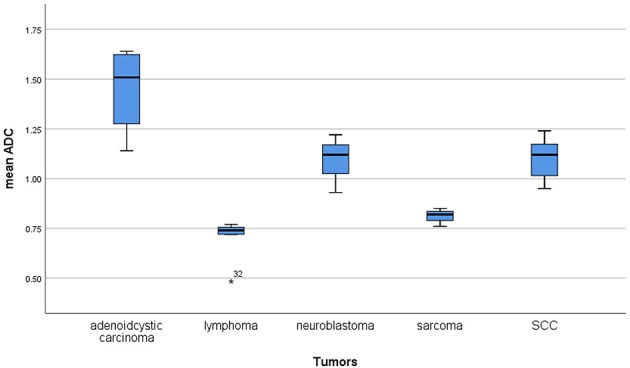
Comparison of apparent diffusion coefficient (ADC) values between different malignant lesions.

Furthermore, the reported mean ADC values of different malignant lesions were analyzed (Figure 6). Lymphomas and sarcomas showed the lowest calculated mean ADC values of 0.7 and 0.79 × 10^−3^ mm^2^/s, respectively. Adenoid cystic carcinomas had the highest ADC values (1.5 × 10^−3^ mm^2^/s). The calculated mean ADC values of squamous cell carcinomas and neuroblastomas were 1.09 and 1.02 × 10^−3^ mm^2^/s, respectively. None of the analyzed malignant tumors had mean ADC values above 1.75 × 10^−3^ mm^2^/s.

## Discussion

Our analysis showed that both malignant and benign lesions in HNR presented with a broad spectrum of ADC values. Although malignant tumors had lower ADC values than had benign lesions, the reported ADC values overlapped significantly. This fact made it impossible to define a reliable threshold for distinguishing malignant and benign lesion in HNR. Furthermore, our finding can explain contradictory results of the previous studies. It is well-known that some benign head/neck lesions, such as cholesteatomas and adenoid hypertrophy, show very low ADC values (36, 37), and some tumors like adenoid cystic carcinomas have high ADC values (8, 9). Presumably, studies with different malignant and/or benign lesions of HNR may have different threshold ADC values. This fact is very important. Therefore, analyses of ADC values between malignant and benign HNR lesions should include all possible entities.

We included all published ADC values of different HNR lesions into the present analysis without selection bias. To the best of our knowledge, our analysis comprises the largest cohort to date. We could not find thresholds in the lower areas of ADC values because malignant and benign lesions overlapped significantly. However, the reported ADC values of all malignant lesions were under 1.75 × 10^−3^ mm^2^/s. Therefore, it may be postulated that lesions with mean ADC values above 1.75 × 10^−3^ mm^2^/s are probably benign. Our results also demonstrated that no real thresholds can be found in the area with <1.75 × 10^−3^ mm^2^/s for the discrimination of malignant and benign lesions. Furthermore, the present analysis showed that lymphomas and sarcomas had the lowest mean ADC values and that adenoid cystic carcinomas had the highest ADC values. This finding is in agreement with that of previous reports (8, 38).

Overall, the present analysis showed that DWI/ADC alone cannot be used as an imaging biomarker of malignancy in the HNR. However, it is known that areas of high cellularity and high proliferation potentially have lower ADC values than have areas of low cellularity, independent of lesion nature (2, 3). Furthermore, numerous previous reports mentioned that necrotic tumor areas show lower ADC values than do solid parts. Therefore, areas of low ADC values may be used as an additional target for biopsies.

Our analysis contains some limitations. Firstly, it is based only on results written in English. Secondly, it analyzed DWI technique using 2 *b* values. However, more advanced imaging techniques, like intravoxel incoherent motion imaging and diffusion kurtosis imaging, which might show a better accuracy in discriminating benign from malignant tumors, were not included in the analysis. Thirdly, we did not analyze a possible influence of some technical details, such as sequence type, choice of *b* values, and Tesla strength. The following aspect should also be addressed: Previously, some authors indicated that ADC values depended significantly on ADC measurements (39, 40). It has been shown that different drawing methods, for example, whole tumor measurements, choice of multiple regions of interest, and/or single region measure, can influence ADC values (39, 40). Therefore, different ADC measurements should be also considered as an important factor. However, a recent large meta-analysis showed that relationships of ADC values between malignant and benign breast lesions were independent of MR technique and measurements (41).

Overall, our analysis is based on heterogenous and predominantly retrospective samples. However, it reflects a real clinical situation in the daily routine.

In conclusion, our analysis showed that ADC values play a limited role in distinguishing between malignant and benign lesions in the HNR.

Lesions with mean ADC values above 1.75 × 10^−3^ mm^2^/s are probably benign. Further large studies are needed for the analysis of the role of DWI/ADC in the discrimination of benign and malignant lesions in the HNR.

## Author Contributions

All authors listed have made a substantial, direct and intellectual contribution to the work, and approved it for publication.

### Conflict of Interest

The authors declare that the research was conducted in the absence of any commercial or financial relationships that could be construed as a potential conflict of interest.

## Abbreviations

HNR: head and neck region
MRI: magnetic resonance imaging
ADC: apparent diffusion coefficient.
